# Repurposing a platelet aggregation inhibitor ticagrelor as an antimicrobial against *Clostridioides difficile*

**DOI:** 10.1038/s41598-020-63199-x

**Published:** 2020-04-16

**Authors:** Matthew Phanchana, Tanaporn Phetruen, Phurt Harnvoravongchai, Ponlawoot Raksat, Puey Ounjai, Surang Chankhamhaengdecha, Tavan Janvilisri

**Affiliations:** 1 Department of Molecular Tropical Medicine and Genetics, Faculty of Tropical Medicine, Mahidol University, Bangkok, 10400 Thailand; 2 Department of Biochemistry, Faculty of Science, Mahidol University, Bangkok, 10400 Thailand; 3 Department of Biology, Faculty of Science, Mahidol University, Bangkok, 10400 Thailand

**Keywords:** Microbiology, Antimicrobials, Antibiotics

## Abstract

Drug resistance in *Clostridioides difficile* becomes a public health concern worldwide, especially as the hypervirulent strains show decreased susceptibility to the first-line antibiotics for *C. difficile* treatment. Therefore, the simultaneous discovery and development of new compounds to fight this pathogen are urgently needed. In order to determinate new drugs active against *C. difficile*, we identified ticagrelor, utilized for the prevention of thrombotic events, as exhibiting potent growth-inhibitory activity against *C. difficile*. Whole-cell growth inhibition assays were performed and compared to vancomycin and metronidazole, followed by determining time-kill kinetics against *C. difficile*. Activities against biofilm formation and spore germination were also evaluated. Leakage analyses and electron microscopy were applied to confirm the disruption of membrane structure. Finally, ticagrelor’s ability to synergize with vancomycin and metronidazole was determined using checkerboard assays. Our data showed that ticagrelor exerted activity with a MIC range of 20–40 µg/mL against *C. difficile*. This compound also exhibited an inhibitory effect on biofilm formation and spore germination. Additionally, ticagrelor did not interact with vancomycin nor metronidazole. Our findings revealed for the first time that ticagrelor could be further developed as a new antimicrobial agent for fighting against *C. difficile*.

## Introduction

A Gram-positive, spore-forming bacterium *Clostridioides difficile*, formerly known as *Clostridium difficile* is currently one of the most concerning nosocomial pathogens^1^. *C. difficile* tops the list of nosocomial infections with the annual estimation of more than 200,000 cases and $1 billion healthcare costs in the United States alone^2^. It has been postulated that *C. difficile* infection (CDI) in humans may come from animals as an overlap between human and animal isolates has been observed^3^. Patients with CDI can appear asymptomatic, however, the excessive use of antibiotics can cause an alteration in indigenous gut microbiota, enabling *C. difficile* to populate, produce toxins and become pathogenic, thereby causing severe diarrhoea, pseudomembranous colitis and occasionally fatality, especially in patients aged more than 65^4^. The treatment for CDI is currently limited to vancomycin, metronidazole, and fidaxomicin^5^, however, increase in treatment failures CDI due to recurrence has rendered these antibiotics ineffective^6^. Although metronidazole was initially used as the first-line agent for treatment of non-severe CDI cases and vancomycin was a drug of choice for more severe and recurrent CDI^4^, however, recent guidelines from the Infectious Diseases Society of America (IDSA) and the Society for Healthcare Epidemiology of America (SHEA) suggests that either vancomycin or fidaxomicin is recommended over metronidazole for an initial episode of CDI, unless in the setting where access to these drugs are limited^5–7^. Emergence of resistant strains to these antibiotics has been demonstrated, causing reduction in the susceptibility^8^. Due to limited treatment options and elevated incidence of drug resistant *C. difficile* strains and treatment failures, the Centers for Disease Control and Prevention (CDC) has listed *C. difficile* as one of the pathogens that poses urgent threats and requires aggressive actions^2^.

**Figure 1:**
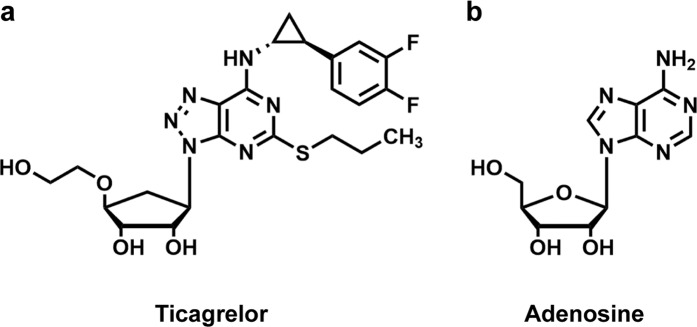
Chemical structures of (**a**) ticagrelor and (**b**) adenine nucleoside.

At present, drug repurposing, or drug repositioning, has gained tremendous attention as an alternative approach for drug development as it shortens the process of drug discovery by nearly a half, as the information on drugs toxicity, pharmacokinetics, and dosages is readily retrievable^9^. A recent report has shown that a nucleoside analog, ticagrelor, functioning as a platelet aggregation inhibitor, which has been approved for prevention of cardiovascular diseases (Fig. 1), displayed antimicrobial activities on various multidrug resistant Gram-positive bacteria but did not affect Gram-negative bacteria^10^. In this study, we further demonstrated the potential of ticagrelor to be repurposed as a novel antimicrobial against *C. difficile in vitro*. We evaluated the effect of ticagrelor on various ribotypes of *C. difficile* in comparison to vancomycin and metronidazole, alone and in combination, and also time-kill kinetics against selected strain. Effects of ticagrelor on biofilm formation and spore germination were also evaluated. Then we investigated further the intracellular components leakage from the bacterial cell by leakage assay and electron microscopy.

## Results

### Ticagrelor exhibited the bactericidal activity against *C. difficile*

We first tested the antimicrobial activity of ticagrelor against different ribotypes of *C. difficile*. Ticagrelor exhibited a MIC of 40 µg/mL for all ribotype tested, with an exception for ribotypes 027 (strain R20291), 106, and 117, which had a MIC of 20–40 µg/mL (Table 1). Although the MICs of ticagrelor were markedly higher than metronidazole and vancomycin, the same range of MICs of ticagrelor was observed in all strains of *C. difficile* tested regardless of their sensitivity background to metronidazole and vancomycin.

**Table 1:** Antimicrobial activity of ticagrelor, metronidazole, and vancomycin against different ribotypes of *C. difficile*.

Ribotype (strain)	Ticagrelor (µg/mL)	Metronidazole (µg/mL)	Vancomycin (µg/mL)
012 (630)	40	0.5	1
017	40	0.5	0.5–1
020	40	0.5	0.5–1
023	40	0.25–0.5	1
027 (R20291)	20–40	0.5	1–2
046	40	0.5	0.5–1
056	40	0.25–0.5	1–2
095	40	0.5–1	0.5–1
106	20–40	0.25–0.5	0.25–0.5
117	20–40	1	1
126	40	1	0.0625–0.125

**Figure 2:**
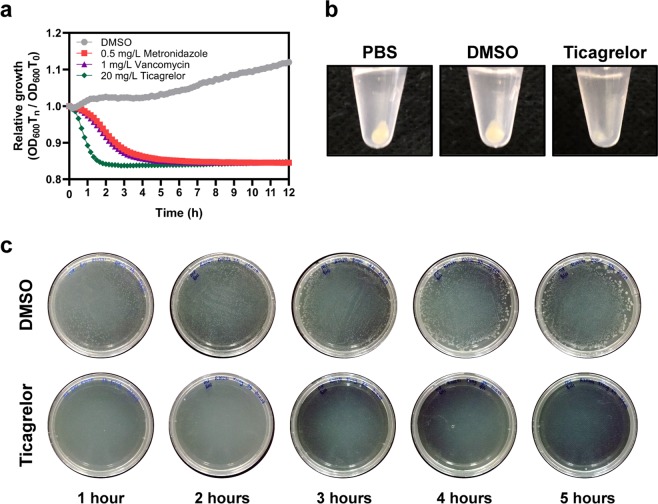
Ticagrelor exhibits a bactericidal activity against *C. difficile*. (**a**) Time-kill kinetics was represented in a relation to the initial OD_600_ over 12 h exposure to various antibiotics; DMSO, circle (•); metronidazole, square (■); vancomycin, triangle (▲); ticagrelor, diamond (♦). (**b**) Bacterial pellets exposed to PBS, DMSO, and 20 μg/mL ticagrelor after 1 h. Intact pellet was barely visible in ticagrelor treatment. (**c**) Bacterial cells exposed to 20 µg/mL ticagrelor and 4% DMSO for 1–5 h were diluted, spread on BHI plate, and incubated for 24 h.

After the antimicrobial activity of ticagrelor was confirmed, we investigated the killing kinetics of ticagrelor with *C. difficile* ribotype 027 (strain R20291). The time-kill kinetics was represented in relative growth, OD_600_ at time n was compared to the initial OD_600_. The results revealed that ticagrelor exhibited a rapid killing profile compared to metronidazole and vancomycin at their respective MIC values (Fig. 2a). Ticagrelor drastically reduced numbers of bacteria after 1 hour (h) incubation, while metronidazole and vancomycin took virtually 5 h to lower the bacterial count to the same level (Fig. 2a). The results from time-kill kinetics were in agreement with the reduced amount of pellet obtained from ticagrelor-treated cells compared to the control (Fig. 2b). Furthermore, there was no viable bacterial count after 24 h incubation even after 1 h exposure to ticagrelor (Fig. 2c). These data thus clearly demonstrated the bactericidal activity of ticagrelor against *C. difficile*.

### Ticagrelor inhibited the formation of biofilm

**Figure 3:**
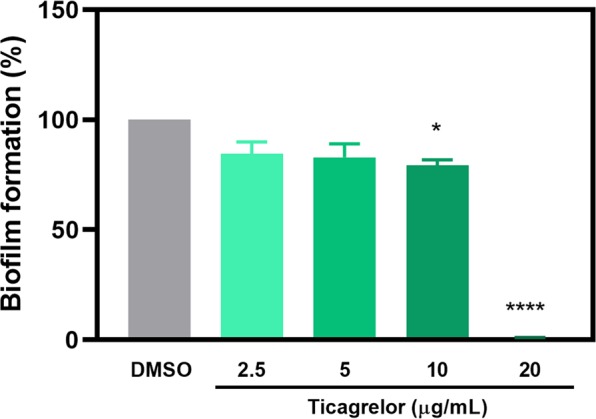
Ticagrelor inhibits biofilm formation in *C. difficile*. Biofilm formation was reduced in sub-MIC treatment with ticagrelor, however it was mostly inhibited when treated with ticagrelor at MIC. Data are presented as mean ± SEM. Bars denoted by (*) and (****) indicate significant difference at p < 0.05 and p < 0.0001, respectively by one-way ANOVA with post-hoc Tukey’s multiple comparison test.

We further investigated the effect of ticagrelor on biofilm formation at the sub-MICs and MIC level with *C. difficile* strain R20291. The production of biofilm at 2.5 and 5 µg/mL ticagrelor treatment was reduced to 85% and 83%, respectively. Statistical analysis revealed no significant difference to the control. Increased concentration of ticagrelor to 10 µg/mL significantly reduced the formation of biofilm to approximately 78% (*p* = 0.022). At 20 µg/mL ticagrelor, the biofilm production was depleted completely (*p* < 0.0001) (Fig. 3).

### Ticagrelor reduced spore germination of *C. difficile*

**Figure 4:**
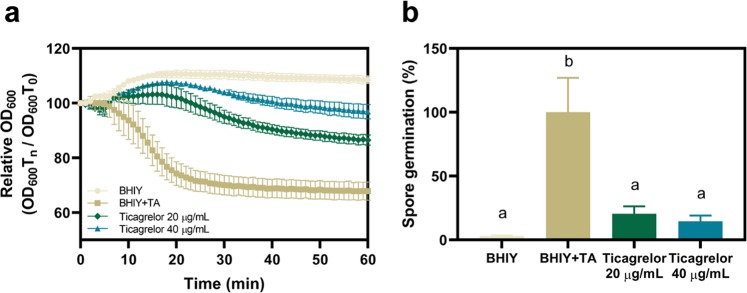
Ticagrelor inhibits *C. difficile* spore germination. (**a**) Spore germination kinetics was presented as the reduction of OD_600_ relative to the initial OD_600_ over 1 h. (**b**) Percentage of spore germination was calculated from the slope of kinetic curve. BHIY, brain heart infusion broth supplemented with 0.5% yeast extract; TA, taurocholic acid. Bars denoted by different letters indicate significant difference by non-parametric ANOVA with post-hoc Dunn’s multiple comparison test.

We tested the effect of ticagrelor on *C. difficile* spore germination using the ribotype 012 (strain 630). Spore germination was measured in a reflection of reduction in OD_600_ over 1 h and represented in relation to the initial OD_600_. Ticagrelor at the MIC level of 20 µg/mL showed strong inhibitory effect on spore germination, up to 80% reduction compared to the control (*p* = 0.0499). Increasing concentration of ticagrelor (to 40 µg/mL) substantially hindered the germination rate, suggesting the dose dependent action of ticagrelor on spore inactivation (Fig. 4).

### Ticagrelor caused leakage of cellular components from the bacterial cells

**Figure 5:**
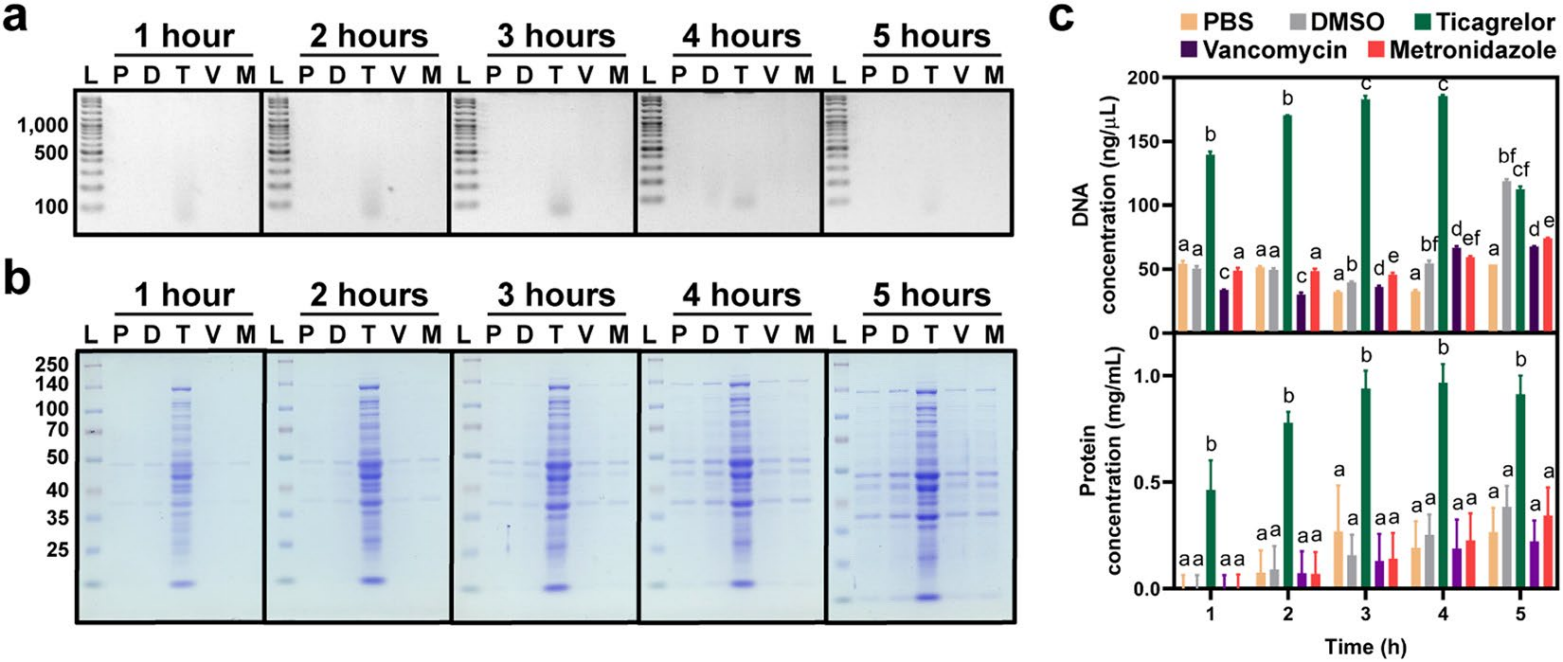
Ticagrelor causes bacterial intracellular component leakage. Leakage assay showed that DNA (**a,c**) and proteins (**b**,**c**) were detected in the supernatant after 1 h incubation with ticagrelor. L, protein ladder; P, PBS; D, DMSO; T, ticagrelor; V, vancomycin; M, metronidazole. Data are presented as mean ± SEM. Bars denoted by different letters indicate significant difference by two-way ANOVA with post-hoc Tukey’s multiple comparison test.

The amount of proteins and DNA leaked from the bacterial cells were measured by Bradford protein assay and NanoDrop spectrophotometer, respectively. Proteins and DNA were detected in the supernatant fraction of ticagrelor-treated *C. difficile* cell culture as early as 1 h after incubation, and increasing over time, while little was observed in other treatment conditions (Fig. 5). The results from gel visualization were in concordant with the quantitative data.

**Figure 6:**
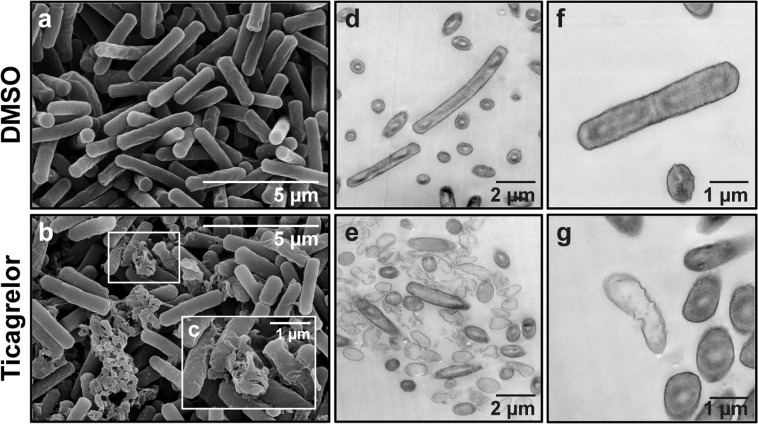
Electron micrographs of *C. difficile* exposed to ticagrelor. Scanning electron micrographs of *C. difficile* exposed to (**a**) 4% DMSO and (**b**,**c**) 80 µg/mL ticagrelor for 2 h. Transmission electron micrograph of *C. difficile* exposed to (**d,f**) 4% DMSO and (**e,g**) 80 µg/mL ticagrelor for 1 h. The scale bars are embedded within the micrographs.

We further investigated the ultrastructure of *C. difficile* by both scanning and transmission electron microscopy (SEM and TEM, respectively). In contrast to the control *C. difficile* cells exposed to DMSO that revealed normal morphology of rod-shaped structure, extensive damages on bacterial cell and leakage of intracellular components were observed in ticagrelor-treated *C. difficile* cells (Fig. 6).

### Ticagrelor has an additive effect to metronidazole or vancomycin

Finally, we evaluated the interaction of ticagrelor with metronidazole and vancomycin. The results showed that ticagrelor has an additive effect to metronidazole and vancomycin by checkerboard assay with the fractional inhibitory concentration index (FICI) falling in a range of 1.5 – 3^11^. It is possible that ticagrelor possesses distinct mode of action compared to metronidazole and vancomycin.

## Discussion

First-line antibiotics for CDI treatment include metronidazole and vancomycin. However, there have been reports on resistance to these drugs, leading to therapeutic failure and poor patient outcome. Therefore, new effective antibiotics are of utmost important in the shadow of lacking approved vaccines^12^. Recently, a report showed that ticagrelor, an approved drug for preventing of thrombotic events in cardiovascular diseases, exhibited antimicrobial activity against several Gram-positive bacteria but not Gram-negative bacteria^10^. As ticagrelor is an FDA-approved drug, development of ticagrelor for CDI falls into drug repurposing approach which could shorten the development process as its pharmacokinetic and safety profiles are readily avilable.

Our data revealed that ticagrelor has a MIC range between 20–40 µg/mL against *C. difficile* (Table 1). These observations are in good accordance with the range previously reported for other Gram-positive bacteria^10^. These findings are also in line with another study that reported the similar MICs of different adenosine analogs against Gram-positive bacteria, which ranged from 16 to 128 µg/mL^13^. Nucleoside analogs have reportedly shown to disrupt membrane function and possess an inhibitory activity for biosynthetic processes including peptidoglycan, cell wall, nucleic acids, folate, and proteins^14^. We hypothesized that ticagrelor might act on the membrane of the bacterium comparable to the known membrane disruptors including nisin and polymyxin B, which have shown to dissipate membrane poteintial^15,17^ – as the killing kinetics was comparable to both membrane disruptors^16,17^ and the bacteriolytic activity was clearly demonstrated (Figs. 2a and 6).

Leakage assays showed that ticagrelor treatment caused bacterial cells to disrupt and release intracellular contents (Fig. 5). These findings support the data from the killing kinetics of ticagrelor, which showed a drastic decrease in cell numbers after 1 h incubation (Fig. 2a). However, leakage of proteins and DNA were also apparent in other treatment conditions at 4 and 5 h post exposure. We hypothesized that this phenomenon is due to the holding capacity of phosphate-buffered saline (PBS) that is incapable to maintain large number of bacterial cells healthy for a prolonged period. Furthermore, deterioration of cellular morphology and membrane surface disruption were observed through both SEM and TEM upon the treatment with ticagrelor, suggesting that ticagrelor exhibited the potent effect against *C. difficile* through cell membrane lysis (Fig. 6). These observations are similar to those of bacteria exposed to TiO_2_, which reportedly causes bacterial cell rupture^18^. However, the exact mechanism how ticagrelor kills *C. difficile* remains to be further explored.

Biofilm formation is one of the features that contributes to pathogenicity of CDI. Solitary *C. difficile* is not highly pathogenic, unless they have aggregated and produced toxins^19,20^. It has been shown that the strains with greater ability to form biofilm are likely to be more virulent^21^. Therefore, the ability of ticagrelor to reduce biofilm formation was evaluated. We showed that ticagrelor at MIC and sub-MIC values reduced biofilm formation. Nevertheless, we speculate that the complete depletion of biofilm at the MIC level of ticagrelor is not likely due to the inhibition of biofilm formation, but rather the inhibition of the bacterial cell growth as there were very little viable cells observed after the treatment. Although biofilm formation in *C. difficile* has been reported to be stimulated by sub-MIC levels of metronidazole^22^ and vancomycin^20^, our data revealed that ticagrelor at the sub-MICs did not induce biofilm formation in *C. difficile*.

Spore is a major transmissive agent in CDI as vegetative cells cannot torelate aerobic environment^23^. Although *C. difficile* spore is naturally resistant to most antibiotics including vancomycin and metronidazole^24,25^, however, in this study, we found that ticagrelor inhibited spore germination in a dose-dependent manner. It has been shown that nucleoside antibiotic derivatives inhibit the outgrowth of *C. difficile* spores at the concentration of 2X MIC^26^. It is possible that nucleoside analogs may compete with the nucleosides required as germinants for *C. difficile* spores^27^. However, the actual mechanism of how nucleoside analogs can disrupt spore germination is still under investigation.

Ticagrelor, formerly known as AZD6140, is a synthetic compound mimicking ATP formulated for an oral administration. It is normally prescribed for prevention of thrombotic events in cardiovascular diseases. As it is an FDA-approved drug, therefore it is relatively safe to use in human. Furthermore, an experiment in murine model reveals low toxicity at the effective dose^10^. However, physiochemical properties showed that ticagrelor has moderate water solubility. In addition, pharmocokinetic data indicate that ticagrelor is poorly absorbed to the circulation with approximately 36% absolute bioavilability^28^ and 84% is excreted, of which 58% through feces and 26% through urine^29^. Considering this information, ticagrelor is deemed suitable for treatment of intestinal pathogens as it fits with the criteria for colon targeting oral drugs^30^. As the dose for human administration approved for cardiovascular disease is about twice of bactericidal concentrations, further investigations are warranted in order to bring this compound forward for drug development. We proposed that the compound should go through the lead optimization process, especially to reduce the binding affinity to its natural receptor P2Y_12_^31^ to reduce the effect of antiplateleting.

## Conclusions

Altogether, we postulated that ticagrelor, an FDA-approved drug for the treatment of acute coronary syndrome, exhibited a bactericidal activity, supposedly with bacteriolytic mode of action against *C. difficile*. Furthermore, ticagrelor also inhibited *C. difficile* biofilm formation and spore germination. Additionally, ticagrelor did not interact with either metronidazole or vancomycin. Ticagrelor could become a promising drug candidate for further development through repurposing approach. However, further investigations are warranted to evaluate the frequency of resistance of *C. difficile* against ticagrelor as well as the effect of ticagrelor in animal models of CDI.

## Methods

### Bacterial culture and minimal inhibitory concentration (MIC) determination

*C. difficile* ribotypes 012 (strain 630), 017, 020, 023, 027 (strain R20291), 029, 046, 056, 095, 106, 117, and 126 were cultured in brain heart infusion (BHI) broth medium supplemented with 0.5% yeast extract (BHIY). Anaerobic condition was provided by anaerobic workstation (Don Whitley Scientific) maintaining at 37 °C. Minimal inhibitory concentration (MIC) assay was performed by microdilution method as per CLSI M11-A6^32^. Briefly, assay plates were pre-filled with 100 µL of various concentrations of test compounds; 0.03–16 µg/mL for metronidazole and vancomycin, 0.15–80 µg/mL for ticagrelor. Ten microliters of 10^7^ CFU/mL bacterial inoculum was then added and incubated for 48 h in anaerobic workstation at 37 °C. Vancomycin solution was prepared in deionized water. Metronidazole and ticagrelor were prepared in DMSO. The assay plates were measured for OD_600_ by a microtiter plate reader (Tecan) to determine the bacterial growth. The MIC value is defined by the lowest concentration of test compound that shows no bacterial growth comparable to blank BHIY medium.

### Time-kill assay

To determine the killing kinetics of the compounds, the time-kill assay was performed with ribotype 027 (strain R20291). Briefly, 100 µL of bacterial inoculum at ~1.5 × 10^8^ CFU/mL was incubated with 0.5 µg/mL metronidazole, 1 µg/mL vancomycin, and 20 µg/mL ticagrelor, then bacterial growth was observed by measuring OD_600_ every 10 min interval for 12 h at 37 °C in a microplate reader (Tecan) under anaerobic conditions. The relative growth was calculated as a ratio of OD_600_ measured at the times T_n_ and T_0_. Cell pellets from DMSO and ticagrelor treatment groups were diluted 100-fold, spread onto BHI agar plates, and incubated for 24 h for viability check.

### Biofilm formation assay

Biofilms of *C. difficile* were generated as mentioned previously with some modifications^33,34^. Briefly, an overnight culture of *C. difficile* strain R20291 was diluted 100-fold into fresh BHIY supplemented with 0.1 M glucose and various concentrations of ticagrelor and incubated in 24-well plate for 48 h at 37 °C. Wells of BHIY without cultures were used as negative controls. To measure biofilm biomass, the cultures were carefully removed from the biofilm plate and wells were washed gently with phosphate-buffered saline (PBS). The biofilms were stained with 0.2% filtered-crystal violet and incubated for 30 min at room temperature. The excess dye was removed from the wells before washing twice with PBS. One milliliter of 1:1 ethanol and acetone solution was added into each well to dissolve dye from biofilm and the absorbance was measured at the wavelength of 570 nm.

### Spore germination assay

*C. difficile* strain 630 was plated on 70:30 sporulation medium and incubated for 5–7 days at 37 °C. To harvest spores, sporulation-induced lawns were collected using distilled water. Spore suspensions were treated with proteinase K, followed by heat treatment at 65 °C for 1 h to eliminate vegetative cells, and washed with distilled water at least 5 times to remove cell debris. Spore purity was confirmed by phase contrast microscopy, and germination test was performed to ensure the viability of the spores. For germination test, prepared spores were heat-activated at 65 °C for 30 min and allowed to cool down on ice. BHI with 0.1% taurocholic acid (TA) was used as a germination medium. Germination kinetics was followed by monitoring the loss of OD_600_ at 1 min interval for 1 h at 37 °C. Germination rate was obtained and calculated from a steepest slope of kinetic plot.

### Leakage assay

Leakage assay was performed to determine the integrity of bacterial cell by observing DNA and protein released from the bacterial cells into supernatant. Briefly, an overnight culture of *C. difficile* strain R20291 was collected and adjusted to OD_600_ of 1.5 with PBS and incubated with 4X MICs of either ticagrelor (80 µg/mL), metronidazole (2 µg/mL), or vancomycin (4 µg/mL) for 5 h at 37 °C. Supernatant and cell pellets were collected every 1 h. Supernatant was used for determination of DNA by agarose gel electrophoresis and NanoDrop spectrophotometer and of protein by Bradford assay and SDS-PAGE. The remaining pellets were further examined by electron microscopy to evaluate the morphology after treatments.

### Scanning electron microscopy

Bacterial cell pellets from leakage assay treated with ticagrelor were observed by scanning electron microscopy (SEM). Briefly, samples were fixed with 4% glutaraldehyde for 24 h, washed twice with 0.5X PBS. Then a series of dehydration in various concentrations of ethanol (50–100%) followed by drying with critical point dryer and platinum/palladium sputtering were applied. Samples were visualized by Hitachi SU8010 field-emission scanning electron microscope (FE-SEM) at an accelerating voltage of 10 kV.

### Transmission electron microscopy

*C. difficile* was treated with either 4% DMSO or 80 µg/mL ticagrelor and incubated for 1 h at 37 °C prior to subjected to transmission electron microscopy (TEM). Bacterial pellets were collected and fixed in 2.5% glutaraldehyde and postfixed in 1% osmium tetroxide for 1 h. Pellets were dehydrated in a series of ethanol ranging from 30 to 100% followed by propylene oxide treatment and finally embedded in Epon epoxy resin. Thin sections of appoximately 90–100 µm were achieved using an ultramicrotome (Leica UC7) and post-stained with 2% uranyl acetate and lead citrate. Samples were imaged using Hitachi HT7700 transmission electron microscope at an accelerating voltage of 100 kV.

### Checkerboard assay

Checkerboard assay was performed to investigate the interaction between ticagrelor and either metronidazole or vancomycin. Two-fold serially diluted ticagrelor ranging from 5–80 µg/mL was mixed with either metronidazole or vancomycin ranging from 0.125–32 µg/mL in 96-well plate. Then 10 µL of bacterial inoculum was added to each well and incubated for 48 h under anaerobic conditions at 37 °C. Endpoint growth was measured by OD_600_. The interpretation of fractional inhibitory concentration index (FICI) value was followed; synergy (≤ 0.5), antagonist (> 4.0), and additive (> 0.5–4.0), while FICI was calculated by FICI = FIC_A_ + FIC_B_, where FIC_A_ = MIC_A+B_/MIC_A_ and FIC_B_ = MIC_B+A_/MIC_B_. MIC_A+B_ is the MIC of compound A in the combination with compound B and vice versa for MIC_B+A_, whereas MIC_A_ or MIC_B_ are the MIC values of the compound alone^11,35^.

### Statistical analysis

GraphPad Prism 8.3.1 was used for statistical analysis. Data from each experiment were tested for normality. Upon passing normality test, data were analyzed by ANOVA with post-hoc Tukey’s multiple comparison test. Otherwise, data were analyzed using non-parametric ANOVA with post-hoc Dunn’s multiple comparison test.

## Data Availability

No datasets were generated or analysed during the current study.
